# One-year Investigator-blind Randomized Multicenter Trial Comparing Asacol 2.4 g Once Daily with 800 mg Three Times Daily for Maintenance of Remission in Ulcerative Colitis

**DOI:** 10.1002/ibd.21938

**Published:** 2011-11-13

**Authors:** A Barney Hawthorne, Rachel Stenson, David Gillespie, Edwin T Swarbrick, Anjan Dhar, Kapil C Kapur, Kerry Hood, Chris SJ Probert

**Affiliations:** * Department of Medicine, University Hospital of WalesCardiff, UK; † SE Wales Trials Unit, School of Medicine, Cardiff UniversityCardiff, UK; ‡ Department of Gastroenterology, New Cross HospitalWolverhampton, UK; § Department of Gastroenterology, Bishop Auckland Hospital, Co.Durham, UK; ‖ Department of Gastroenterology, Barnsley District General HospitalBarnsley, UK; ¶ Department of GastroenterologyLiverpool University, UK

**Keywords:** mesalazine, ulcerative colitis, adherence, clinical trial

## Abstract

**Background:**

Mesalazine (Asacol) is still widely prescribed in divided doses for ulcerative colitis (UC), despite evidence that adherence is improved by once-daily (OD) prescribing. We aimed to investigate whether OD Asacol was as effective as three times (TDS) daily dosing, and to evaluate the role of treatment adherence.

**Methods:**

An investigator-blind randomized trial was undertaken comparing OD Asacol (three 800 mg tablets) versus one 800 mg TDS in maintenance of remission of UC over 1 year. The primary endpoint was relapse rate, and noninferiority would be concluded if the lower limit of the two-sided 95% confidence interval (CI) of the difference in proportions relapsing (TDS-OD) exceeded −10%. Adherence was measured by tablet counts and self-reported adherence. A subgroup of patients used a bottle cap that recorded all bottle opening events.

**Results:**

In all, 213 patients were randomized. In the intention-to-treat (ITT) population, relapse rates were 31% (95% CI 22%–40%) in the OD and 45% (95% CI 35%–54%) in the TDS group. Primary analysis confirmed the noninferiority of OD dosing. Two of the study populations, ITT and per-protocol (PP), showed potential superiority of OD dosing. All measures of adherence showed that it was significantly better in the OD group. Multivariate analysis, however, showed OD dosing was associated with lower relapse risk independently of adherence.

**Conclusions:**

OD dosing with Asacol 2.4 g is as safe and effective as TDS dosing, and secondary analysis confirmed significantly reduced relapse rates. The benefit, however, was clinically borderline and may relate in part to ease of adherence. (Inflamm Bowel Dis 2012)

Coated formulations of mesalazine (Asacol) have been demonstrated in many trials to prevent relapses of ulcerative colitis (UC) in patients who have achieved remission.^1^ Treatment adherence remains a major limitation to treatment success and, until recently, all trials have used divided dosing schedules. This dates back to the use of sulfasalazine, which was given in split dosage in order to reduce the likelihood of toxicity from sulfapyridine. Trials of other azo-bonded molecules (olsalazine and balsalazide) and coated mesalazine preparations used similar divided doses, varying from twice to four times daily dosing. Most trials of Asacol (Warner Chilcott Pharmaceuticals, Dublin, Ireland) used three times daily dosing schedules (TDS). The dosing schedule has a major impact on patient adherence,^2^ particularly for TDS dosing,^3^ and there is increasing interest in evaluating once-daily (OD) dosing of mesalazine. It is reported that in normal subjects, median peak concentrations, trough concentrations, and areas under the curve were similar for OD and TDS Asacol dosing regimens.^4^ There is also evidence to show that patients with quiescent UC have colonic motility and function that is similar to healthy controls.^5^,^6^ The pharmacokinetics of mesalazine should therefore be the same in quiescent UC as in the normal colon. However, it is generally considered that the mechanism of action of mesalazine is exerted topically, and thus plasma levels may have more relevance for toxicity than efficacy.^7^ A pilot feasibility study of OD (Asacol 400 mg) versus conventional dosing of mesalazine for the maintenance of remission of UC gave similar outcomes, but the authors concluded that a larger trial was warranted.^8^

The CODA study (Colitis Once Daily Asacol) was designed to compare OD mesalazine given as three 800 mg Asacol tablets, in comparison to one Asacol 800 mg tablet given TDS, as maintenance therapy for UC over 1 year. The trial objectives were to evaluate whether OD dosing was as effective as TDS dosing, and as safe. A substudy was designed to evaluate the impact of OD dosing on treatment adherence.

## MATERIALS AND METHODS

### Trial Design

The study was an investigator-blind, multicenter trial comparing OD Asacol given as three 800 mg tablets (OD group), to one 800 mg Asacol tablet given three times daily (TDS group) as a maintenance therapy over a 1-year period or until relapse of UC.

### Participants

Patients were recruited with UC in remission on maintenance therapy with mesalazine, sulfasalazine, olsalazine, or balsalazide for at least 4 weeks, but who had had at least one relapse within the previous 2 years. Patients had to be aged over 18, if female to be taking adequate contraception (if otherwise able to conceive), and able to give informed consent. Patients were excluded if they had Crohn's disease; symptoms of active colitis; a modified Baron^9^ score (see below) at sigmoidoscopy of 2 or 3; used enema or suppository therapy for UC in the past 4 weeks; had started or altered the dose of azathioprine or 6-mercaptopurine in the past 3 months (these drugs were permitted if in stable dosage over that period of time); had intolerance to mesalazine; known HIV infection; significant renal or hepatic impairment; or other medical or psychiatric disorder (including alcohol dependence) that in the opinion of the investigator would affect participation in the study; or females if pregnant or lactating.

### Initial Evaluation

At the screening visit, eligible patients gave written, informed consent; blood was taken for urea, creatinine, and C-reactive protein; urine was analyzed for blood and protein by dipstick; and flexible or rigid sigmoidoscopy was performed. Mucosal inflammation was graded using a modification^10^ of the Baron score (grade 0: normal appearance; grade 1: erythema, decreased vascular pattern; grade 2: marked erythema, absent vascular pattern, friability, erosions; grade 3: spontaneous bleeding, ulceration). Symptoms of disease activity were assessed using the Mayo score,^11^ with the full score incorporating the modified sigmoidoscopy score. Details of the patient's UC, other medical history, and concomitant medication were obtained. The baseline visit was scheduled for up to 7 days later, but could take place on the same day as screening, if results of urea and creatinine were available. At baseline a stool sample was provided for measurement of fecal calprotectin; samples were posted to the central laboratory (University Hospital of Wales, Cardiff, UK) where they were frozen at −20°C for batched analysis. Calprotectin results were not made available to clinicians during the study.

### Randomization

Patients were randomized to OD or TDS in a 1:1 ratio. Randomization was carried out in advance within the South East Wales Trials Unit, who generated sequence codes to allocate patients to either group (kept in each center's trial pharmacy in opaque, sequentially numbered, sealed envelopes). Centers were stratified and allocation was carried out using random permuted blocks of size four or six (randomly selected). Trial pharmacists issued patients with trial medication bottles, with labels attached indicating the treatment regimen to which they had been allocated. The study was investigator-blind and patients were instructed not to reveal to either research nurse or doctor which treatment group they were in. This advice was repeated at the start of each study visit.

### Follow-up Evaluations

At 6-week, 6-month, 12-month (final) clinic visits, or in the event of suspected relapse, disease was assessed using the stool frequency and rectal bleeding component of the Mayo Clinic score. Patients were asked about treatment adherence, adverse events, had count of returned tablets, blood test for renal function, and urinalysis for blood and protein. If patients had symptoms of relapse they were instructed to attend the clinic urgently for assessment. At the final (or relapse assessment) visit, patients also underwent rigid or flexible sigmoidoscopy and a Mayo score was calculated. In addition to the clinic visits, telephone follow-up was carried out at 3 and 9 months to record adverse events and changes to concomitant medication.

### Outcomes

The primary endpoint of the study was the relapse rate during the 12-month follow-up. Relapse was defined as symptoms of active disease (bloody diarrhea or rectal bleeding for 3 days or more) with a sigmoidoscopic appearance of grade 2 or 3 using the modified Baron score. If patients were inadvertently treated for active disease before returning to the clinic, they were also deemed to have relapsed. Secondary outcomes included time (in weeks) until confirmed relapse and analysis of factors (time since last relapse before trial entry, concomitant medication, disease extent and duration, smoking status, age at diagnosis, baseline sigmoidoscopy score, and calprotectin level) as effect modifiers. Analysis of adverse events and adherence to treatment was also performed.

### Sample Size

Analysis of 14 trials of maintenance therapy with mesalazine of 1 year duration show great variability, with relapse rates from 23%–63%.^12^–^24^ The mean relapse rate of these pooled trials (involving 1540 patients) was 42%. A major determinant of relapse rate is the length of previous remission,^25^ and many of these trials required a shorter time since last relapse before entry. With a criterion of relapse within the past 2 years, the estimated relapse rate was 20%–30%. A difference of 10% between treatment groups was considered the minimum clinically important effect. A total of 250 patients were needed to demonstrate noninferiority (a difference of less than 10%) with a one-sided α value of 5%, and power of 80%.

### Adherence Substudy

A subgroup of patients was invited to participate in a substudy, with a separate consent process. These patients were given a bottle cap to record adherence throughout the study. The Medication Event Monitoring System (MEMS) based in this bottle cap recorded each time the bottle was opened and data were uploaded onto a database at each trial visit. Bottle opening (dosage event) was assumed to equate to tablet consumption. Analysis was performed on the complete case population. Daily adherence was defined for OD patients as one dosage event (or more) within 24 hours, and for TDS patients as three dosage events (or more), separated each by more than 1 hour.

### Statistical Methods

The primary analysis was a noninferiority test of the OD versus TDS regimen, comparing the proportion of patients relapsing by 12 months in the two treatment groups. A two-sided 95% confidence interval (CI) for the difference in proportion of patients relapsing (calculated as TDS – OD) was calculated. Noninferiority was concluded if the lower limit of the 95% CI was more than −10% in the complete case (CC) population (the intention-to-treat [ITT] population for whom primary outcome data could be obtained), the ITT population (imputing all patients with missing outcome data (lost to follow-up or withdrawn for reason other than relapse as relapsed), and the per-protocol (PP) population (the CC population who had adherence of at least 75% and who met all inclusion and exclusion criteria at trial entry).

Secondary analysis included a superiority analysis of the primary endpoint (if noninferiority was shown in CC, ITT, and PP populations), comparison of median time to relapse using Kaplan–Meier methods, a noninferiority test of the number of adverse events, treatment adherence, and analysis of potential interactions using a multivariate logistic regression model. Center effects were tested for, but left out of the final model, if they were not significant. Medication adherence was investigated by comparing the mean daily dose (based on tablet counts) with the expected dose. Patients were considered adherent if they took at least 75% of the expected dose. For the adherence substudy, cofactors including treatment group, age, sex, employment status, and relapse/nonrelapse were explored in a multivariate regression model. Data were analyzed using SPSS v. 16.0 (Chicago, IL).^26^

### Ethical Considerations

The study was performed in accordance with the principles of Good Clinical Practice and the Declaration of Helsinki and received ethical approval. Written informed consent was obtained from each patient. The study was registered at ClinicalTrials.gov (NCT00708656).

## RESULTS

### Patient Population

Between 2006 and 2009, 32 UK centers assessed 579 patients for inclusion in the study, and 213 patients were randomized (103 to the OD group, 110 to the TDS group) and formed the ITT population. All received trial medication. The two groups were well-matched, as shown in Table 1. The average age at entry was 50 years, and 30% had extensive colitis, 55% left-sided colitis or proctosigmoiditis, and 14% had proctitis. The median duration of remission prior to entry was 6 months. At entry, the median fecal calprotectin was 62.1 mg/kg stool (OD group) and 89 mg/kg stool (TDS group), (*P* = 0.048); 51.5% (OD group) and 61.9% (TDS group) had a baseline calprotectin level above 60 mg/kg stool (*P* = NS). Prior to entry, about three-quarters of patients were taking Asacol, and most patients entering the study were taking their 5-aminosalicylate (5-ASA) in twice or TDS dosage (Table 1). None were taking infliximab therapy.

**TABLE 1:** Baseline Demographics (No Significant Difference Between Groups Excepting Calprotectin, *P* = 0.048)

		Once Daily (*n* = 103)	Three Times Daily (*n* = 110)	All Patients
	Age^a^	49.5 (15.0)	50.0 (14.9)	50.4 (14.9)
Gender^b^	Male	51.5 (53)	50.0 (55)	50.7 (108)
Maximum documented extent of UC^b^	Extensive	30.1 (31)	30.8 (33)	30.0 (64)
	Left-sided or sigmoid	61.2 (63)	50.5 (54)	54.9 (117)
	Proctitis	8.7 (9)	18.7 (20)	13.6 (29)
Smoking status^b^	Nonsmoker	46.6 (48)	46.4 (51)	46.5 (99)
	Current smoker	6.8 (7)	14.5 (16)	10.8 (23)
	Exsmoker	46.6 (48)	39.1 (43)	42.7 (91)
Employment status^b^	In full-time employment	51.5 (53)	49.1 (54)	50.2 (107)
	Not in full-time employment	48.5 (50)	50.9 (56)	49.8 (106)
	Disease duration (years)^c^	3.0 (1.0, 10.0)	5.0 (2.0, 13.0)	4.0 (1.5, 12.5)
	Number of relapses in past two years^c^	1.0 (1.0, 2.0)	1.0 (1.0, 2.0)	1.0 (1.0, 2.0)
	Duration of remission (months)^c^	6.0 (3.0, 11.5)	6.0 (3.0, 13.0)	6.0 (3.0, 12.0)
	Calprotectin result (mg/kg stool)^c^	62.1 (20.3, 120.4)	89.0 (32.0, 180.6)	78.0 (23.3, 159.4)
Baseline sigmoidoscopic score^b^	Normal (0)	76.7 (79)	65.5 (72)	70.9 (151)
	Not normal (1)	23.3 (24)	34.5 (38)	29.1 (62)
Baseline 5-aminosalicylic acid medication^b^	Asacol	75.7 (78)	73.6 (81)	74.6 (159)
	Pentasa	13.6 (14)	11.8 (13)	12.7 (27)
	Balsalazide	5.8 (6)	8.2 (9)	7.0 (15)
	Other	4.8 (5)	6.3 (7)	5.6 (12)
Baseline 5-aminosalicylic acid dose frequency	Once	7.9 (8)	7.3 (8)	7.5 (16)
	Twice	47.5 (48)	52.3 (57)	49.3 (105)
	Three times	43.6 (44)	40.4 (44)	41.3 (88)
	Four times	1.0 (1)	0 (0)	0.5 (1)
	Azathioprine or 6-mercaptopurine use^b^	10.7 (11)	12.7 (14)	11.7 (25)

a Mean (standard deviation).

b Percentage (number of patients).

c Median (interquartile range).

### Patient Disposition

**FIGURE 1:**
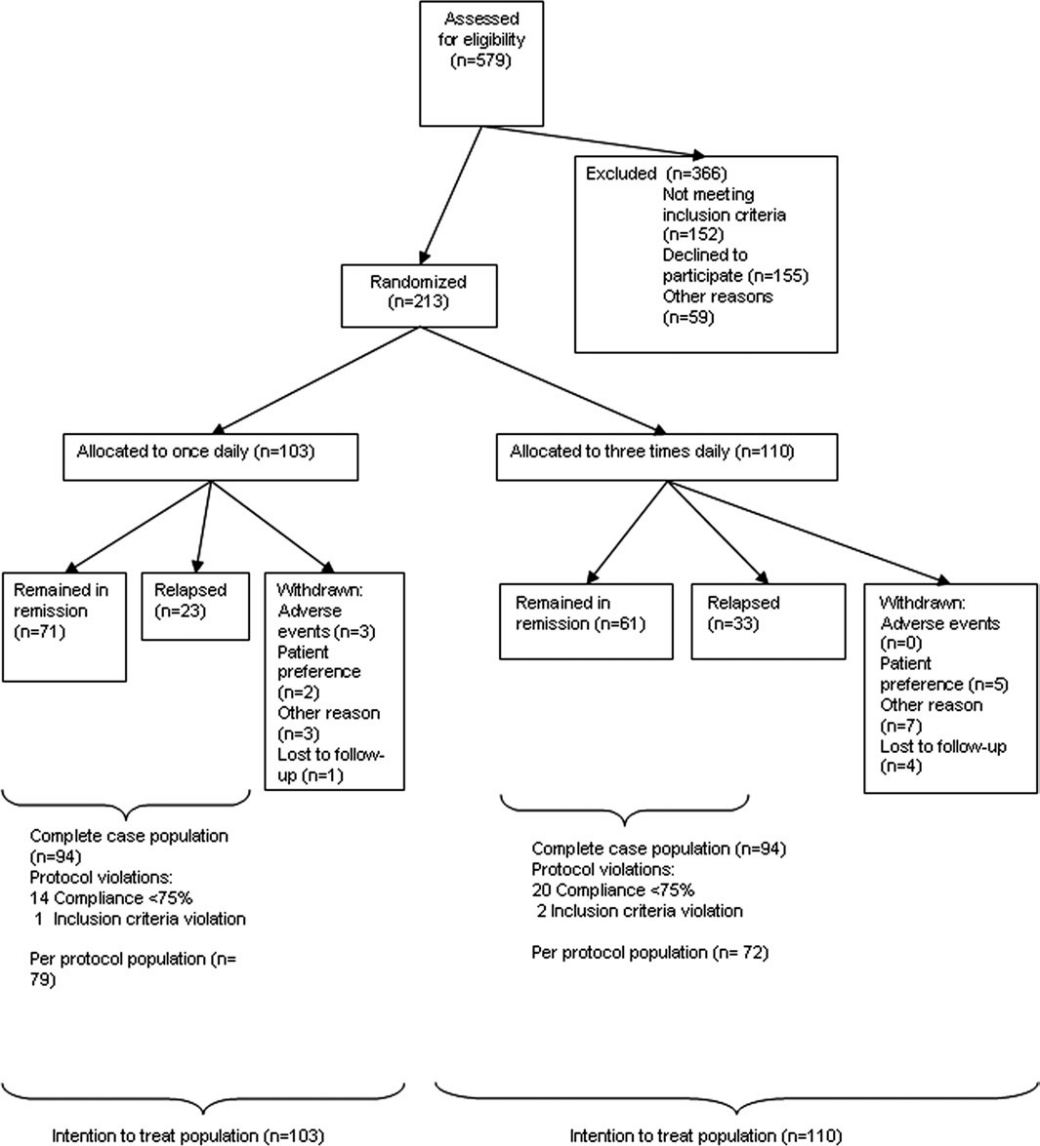
Patient disposition in trial.

The flow of patients in the study is shown in Figure 1. Nine patients (9%) of the OD group and 16 (14.5%) of the TDS group were withdrawn for reasons other than relapse, leaving 94 in each group forming the CC population. Fifteen patients in the OD group had protocol violations (14 adherence less than 75% [or data missing], one inclusion criteria not fulfilled), leaving 79 patients in the PP population. In the TDS group, 22 patients were not included (20 adherence less than 75% [or missing data] and two inclusion criteria violation) leaving 72 patients in the PP population.

### Efficacy Analysis

**FIGURE 2:**
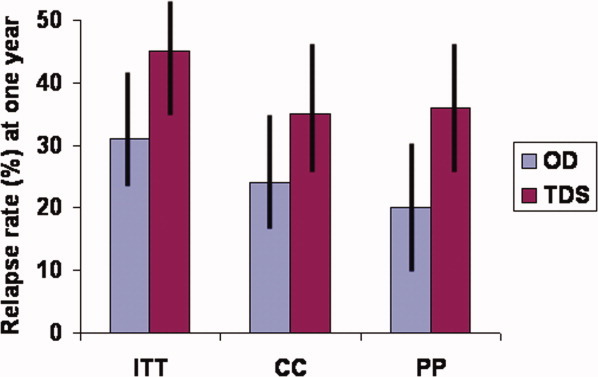
Relapse rates in the ITT, CC, and PP populations. OD group given 2.4 g mesalazine once daily (three 800 mg Asacol tablets); TDS group given 800 mg tablet three times a day. Shown as percentage and 95% confidence intervals. [Color figure can be viewed in the online issue, which is available at wileyonlinelibrary.com.]

Relapse rates were 31% (95% CI 22%–40%) in the OD and 45% (95% CI 35%–54%) in the TDS group for the ITT population (Fig. 2). In the PP population relapse rates were 20% (95% CI 11%–29%) in the OD and 36% (95% CI 25%–47%) in the TDS group. In the CC population the relapse rate was 24% (95% CI 16%–33%) in the OD and 35% (95% CI 25%–45%) in the TDS group. The difference between relapse rates (TDS – OD) was 14% (95% CI 1%–26%) (ITT population), 16% (95% CI 2%–30%) (PP population), and 11% (95% CI −2%–24%) (CC population). As the lower limit of the confidence interval was not less than −10% in any of the three populations, noninferiority of the OD therapy was confirmed. Two of the three study populations indicate a potential superiority of OD over TDS dosing.

**FIGURE 3:**
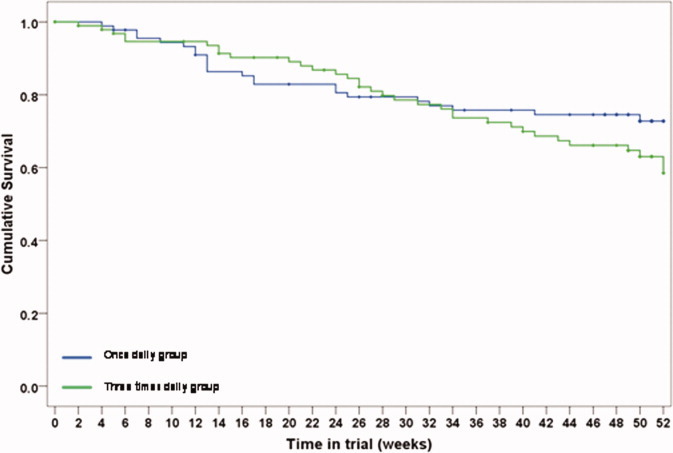
Remission rate in the once daily group (blue line) versus the TDS group (green line). *P* = 0.211 (log rank, Mantel-Cox). [Color figure can be viewed in the online issue, which is available at wileyonlinelibrary.com.]

The Kaplan–Meier plot of remission rate is shown in Figure 3: the difference between the two treatments was nonsignificant in this analysis. In a multivariate analysis of factors affecting likelihood of relapse (Table 2), TDS treatment group, being an exsmoker, and baseline sigmoidoscopic score of 1 were independent predictors of relapse (per protocol population) with odds ratio (OR) >1 (i.e. relapse more likely). An abnormal baseline calprotectin value (>60 mg/kg stool) was not an independent predictor of relapse, although baseline calprotectin was significantly higher in the 56 patients with a sigmoidoscopy score of 1 (107.9 [36.4–346.2]) (median [IQR]) than those with a score of 0 (62.1 [22.0–119.5], *P* = 0.001).

**TABLE 2:** Multivariate Logistic Regression Analysis of Relapse Using Complete Case Population (*n* = 153)

	Odds Ratio	95% CI	*P*-value
Intercept	0.33	0.03–4.22	0.392
Once daily group	1.00		
Three times daily group	2.51	1.09–5.76	0.031
Age at baseline (decade increase)	0.73	0.53–1.01	0.059
Male	1.00		
Female	0.62	0.26–1.48	0.278
Never smoked	1.00		
Current smoker	0.64	0.11–3.86	0.628
Exsmoker	2.61	1.05–6.47	0.039
Not in full-time employment	1.00		
In full-time employment	0.98	0.39–2.46	0.959
Baseline sigmoidoscopy score 0	1.00		
Baseline sigmoidoscopy score 1	3.37	1.47–7.76	0.004
Baseline calprotectin ≤60mg/kg	1.00		
Baseline calprotectin >60mg/kg	1.53	0.66–3.58	0.324
Duration remission <1 yr prior to entry	1.00		
Duration remission ≥1 yr prior to entry	0.40	0.14–1.12	0.081
<75% of prescribed dose	1.00		
≥75% of prescribed dose	1.75	0.30–10.10	0.534

### Treatment Adherence in Main Study

According to tablet counts, the average daily number of tablets taken during the trial was 2.93 (2.8–3.0) (median and IQR) for the OD group (*n* = 84), versus 2.79 (2.56–3.0) for the TDS group (*n* = 80) (*P* = 0.005). This excludes 49 missing items of tablet count data. 95.2% patients were more than 75% adherent in the OD group, compared to 92.5% in the TDS group (*P* = 0.46). Patients were also asked how easy it was to remember to take their tablets with their allocated regimen. In the OD group, 81.5% found it very easy, 16.3% fairly easy, 2.2% fairly difficult, and none very difficult. Figures in the TDS group were 34% (very easy), 39.4% (fairly easy), 21.3% (fairly difficult), and 5.3% very difficult (*P* < 0.001 vs. OD group). In a multivariate analysis, adherence based on tablet count was not an independent predictor of relapse (Table 2), nor was the interaction factor between categorized adherence and treatment group significant (data not shown). Patients were asked (at their final visit) whether they had taken their drugs as prescribed at least 90% of the time, and 96.8% (OD group) answered “yes” compared to 85.1% (TDS group) (*P* = 0.012). Using this perceived adherence in the multivariate model did not again demonstrate a significant impact of adherence in the likelihood of relapse.

### Adverse Events

Overall, 83 events were reported in the OD group, 85 in the TDS group. The commonest adverse events were gastrointestinal in both groups. Adverse events were considered probably or definitely related to trial medication in two (OD group) and one (TDS group) instances. Serious adverse events in the OD group were abdominal pain, five admissions for surgery, and one worsening of UC. Serious adverse events in the TDS group were two hospital admissions, unrelated to UC or trial medication. There was no difference in numbers reporting one or more adverse events (OD group 47%, TDS group 44%). Baseline creatinine was 81.3 (14.5) μmol/L in OD group (mean [standard deviation]), and 81.8 (15.3) μmol/L in the TDS group. The change from baseline to final visit was −0.05 (−1.8 to 1.7) μmol/L (mean and 95% CI) in the OD group, and −1.8 (−3.8 to 0.28) μmol/L in the TDS group. No deaths occurred during the study.

### Adherence Substudy

Fifty-eight patients were entered into the substudy (28 in OD group, 30 in TDS group). All patients in Cardiff and Bristol in the main study were asked to join the substudy.

**FIGURE 4:**
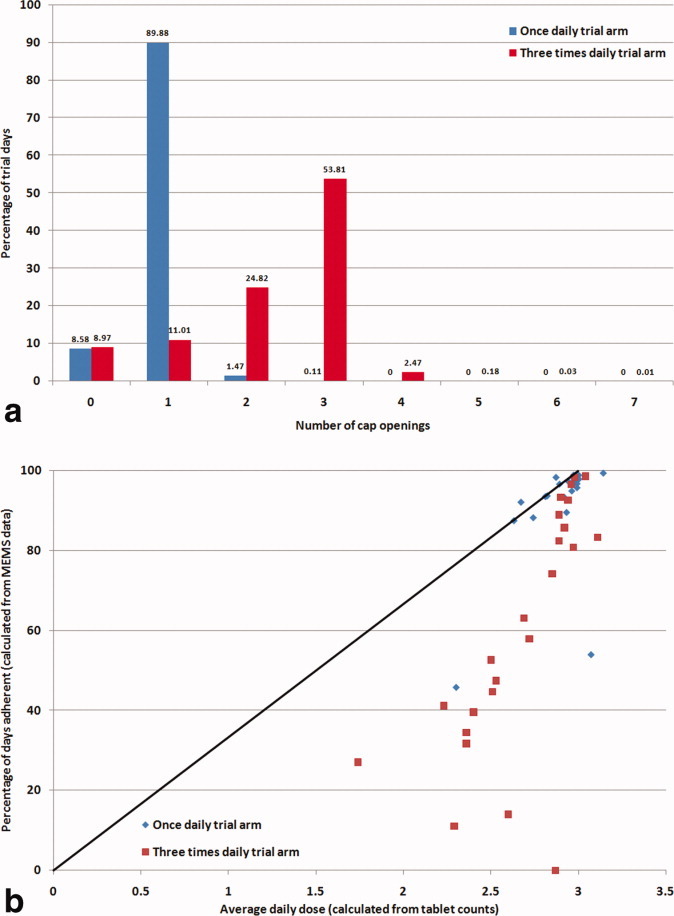
(a) Frequency plot of number of cap openings in 1 day during trial (substudy population, ITT group). (b) Percentage days adherent as measured by MEMS data, plotted against mean daily tablets taken measured by tablet count. Line represents equivalence (substudy population, ITT group). [Color figure can be viewed in the online issue, which is available at wileyonlinelibrary.com.]

Treatment adherence was significantly better in the OD group, with median days adherent (opening cap once or more) 96.6% (IQR 92.7%–98%), versus a median 54.8% (IQR 34.4%–85.7%) days in the TDS group when the cap was opened at least three times (*P* < 0.001). Treatment adherence was not significantly affected by patient age, gender, treatment center, or employment status. Tablet counts and treatment adherence based on cap opening data correlated closely (R = 0.738, *P* < 0.001). Patients' perception of their adherence was also corroborated by cap opening. Patients stating they were at least 90% adherent (*n* = 50) were adherent on cap opening data on a median 92.9% days (IQR 63.1%–97.3%), compared to a median 34.1% days (IQR 11.3%–45.8%) for those stating they were less than 90% adherent (*n* = 6). Similarly, patients stating that adherence to taking trial medication was easy (*n* = 27) were adherent on cap opening data on a median 96.7% days (IQR 92.7%–98.0%) compared to those who stated it was difficult (*n* = 10) who were adherent on a median 46.6% days (IQR 40.0%–57.8%). The frequency of cap opening, according to treatment group, is shown in Figure 4a, and illustrates that, while poor adherence usually means under-dosing, there was occasional extra opening of bottles in both the OD and TDS groups. A plot of percentage of days adherent using cap opening against daily dose according to tablet counts data shows that tablet count data produces an overestimate of adherence levels in all patients where it is low (Fig. 4b).

Three patients in the OD group relapsed (11.1%) versus 13 patients in the TDS group (44.8%). The difference in relapse rate (complete case population) was 33% (95% CI 12%–55%). In a regression model of relapse incorporating treatment group and percentage days adherence, TDS treatment group was significantly associated with relapse (OR 7.1, 95% CI 1.4–35.6, *P* = 0.017) but patients being adherent for at least 75% of the days they participated in the trial did not significantly affect likelihood of relapse (Table 3).

**TABLE 3:** Multivariate Logistic Regression Model of Relapse Using the Complete Case Population in the Substudy (*n* = 56)

Population: Complete Case (*n* = 56)				

	Odds Ratio	Lower 95%	Upper 95%	*P*-value
Intercept	0.11	0.02	0.66	0.016
Once daily	Reference category for trial arm			
Three times daily	7.08	1.41	35.58	0.017
Days adherent less than 75%	Reference category for percentage of days adherent			
Days adherent at least 75%	1.17	0.28	4.93	0.831

Days adherent defined as opening cap once or more on that day (OD group) or three times or more on that day (TDS group).

## DISCUSSION

This study has demonstrated that Eudragit-S coated mesalazine (Asacol) at a daily dose of 2.4 g is at least as effective when given OD, compared to TDS dosing. The primary endpoint of noninferiority was met for both the ITT and PP populations, and a secondary analysis showed a statistical superiority of OD dosing.

The study was a single-blind design so that the important aspect of patient adherence could influence the outcomes and it was hoped that a 1-year duration would give a more realistic approximation to normal patient behavior, as well-motivated patients entering clinical trials generally have better adherence.^27^ A TDS dosing was chosen as the comparator group, because this is the most logical way to take three tablets of 800 mg in divided doses and significant numbers of gastroenterologists still prescribe TDS dosing. (In September 2010, an on-line survey in the UK showed that 25% of patients were still prescribed TDS Asacol, Warner Chilcott, data on file.) 33% of North American patients were taking TDS mesalazine prior to entering the QDIEM study of OD mesalazine (2007–2009).^28^

The two treatment groups were well-matched at baseline. The baseline sigmoidoscopy score was an independent predictor of relapse, but baseline calprotectin level was not, in contrast to other studies showing good predictive value for relapse in UC,^29^,^30^ although these studies did not compare the value of endoscopic assessment. Smoking status was also independently predictive of relapse, with exsmokers at increased risk (OR 2.6, 95% CI 1.0–6.9) versus nonsmokers, and compared to a modest protective effect in current smokers. Costa et al^29^ also found that exsmokers were at highest risk of relapse in UC.

One of the strengths of this study is the detailed adherence data provided by the MEMS caps results. Most patients in Cardiff and Bristol entered the substudy, and for the few who did not the main reason was the inconvenience of carrying a large bottle, with the bulky cap during the day, as they would have to if recruited to the TDS group. It is well recognized that adherence is significantly worse for any drug regimen of TDS, compared to twice daily, whereas the adherence difference is less dramatic between twice and OD.^31^ The cap opening data gave objective evidence that adherence was significantly better in the OD group (after making the assumption that patients actually ingested the tablets in the correct dose after opening the bottle). The lack of influence of other patient factors on adherence in the substudy (including age, sex, and employment status) may be attributable to the limited sample size, as other studies have shown that these have a significant impact on adherence.^27^ Cap opening also correlated closely with the tablet count data in the substudy (Fig. 4b), but provides confirmation that tablet count data consistently overestimates adherence in those with poor adherence. As in other studies, patients self-reported adherence was confirmed by cap opening data (self-reporting of adherence correlates with urinary 5-ASA).^2^,^3^ The apparent lack of influence of adherence on likelihood of relapse in either the main study or the substudy was unexpected. In the main study, this may be explained by the inaccuracy of tablet count data as a measure of adherence and sample size would account for lack of significance in both main and substudy. In addition, it is plausible that the relatively high dose of 2.4 g daily dose may have provided some leeway for under-dosing caused by nonadherence, if a lower dose was sufficient to keep most patients in remission.

Since this trial was started, a number of other studies have evaluated OD mesalazine formulations. The QDIEM study^28^ also evaluated Asacol. This large investigator-blind study compared once- with twice-daily dosing, but allowed patients to continue their usual daily dose, which had to be between 1.6 and 2.4 g. Relapse rates were low (less than 15% at 1 year). Adherence was measured using the MARS medication adherence questionnaire,^32^ but without tablet counts, and the only significant difference in reported adherence was at 3 months (in favor of OD) but was very high throughout the study. The only other maintenance trial of tablet mesalazine was the MMX mesalazine study^33^ and showed no difference in 1-year relapse rates, with high adherence in both once- and twice-daily arms. In comparison with these studies, our study had significantly lower adherence in the TDS group, and this still remains the most plausible explanation for the difference in relapse rates.

Two studies have evaluated maintenance therapy with mesalazine granules. The PODIUM study of Pentasa ethyl cellulose coated granules^34^ did show lower relapse rates for OD therapy compared to BD, and although sachet counts did not detect a difference in adherence, the visual analog score for adherence for the OD group was significantly better at visits 2 and 3 (compared to BD) but not at trial end. The OD maintenance study of Salofalk granules (Eudragit-L coated mesalazine)^35^ compared 3 g OD with 1.5 g OD and 500 mg TDS, with lower relapse rate for 3 g OD, and numerically lower relapse rate for 500 mg TDS than 1.5 g OD. It was not possible to assess adherence as the study was double-blind, double-dummy.

There is no perfect way to measure treatment adherence. The MEMS data provide accurate information on cap opening, but we have to assume that when the cap is opened the tablets are actually taken out and ingested, in the correct number. It is also possible that TDS patients forgetting their midday dose may have taken two tablets as their evening dose. Many patients would have preferred to transfer their midday tablets to a smaller container in the morning, to take to work and consume in the middle of the day. The use of the cap meant that patients had to carry the whole pill container with them (capacity for 180 tablets), and this significantly disadvantaged the TDS group. This was the commonest reason for patients not wishing to enroll to the substudy. Our study is also limited by sample size. Recruitment was slow, and the study was terminated as data emerged from other OD studies.

This study provides confirmatory evidence that OD dosing is as effective as divided doses, and it is now clear that this applies to all mesalazine formulations. While statistically superior to TDS therapy, the difference in relapse rates was not sufficiently large to confirm clinical superiority, as the confidence intervals for the difference crossed the prespecified 10% gap that was deemed clinically meaningful. Adherence was significantly better in the OD group compared to TDS throughout the 1-year study, and still remains the most likely explanation for the difference in favor of OD therapy.
